# ForestSubtype: a cancer subtype identifying approach based on high-dimensional genomic data and a parallel random forest

**DOI:** 10.1186/s12859-023-05412-y

**Published:** 2023-07-19

**Authors:** Junwei Luo, Yading Feng, Xuyang Wu, Ruimin Li, Jiawei Shi, Wenjing Chang, Junfeng Wang

**Affiliations:** School of Software, Henan Polytechnic University, Jiaozuo, China

**Keywords:** Cancer subtyping, Random forest, Gene expression data, Machine learning, Auto Encoder

## Abstract

**Background:**

Cancer subtype classification is helpful for personalized cancer treatment. Although, some approaches have been developed to classifying caner subtype based on high dimensional gene expression data, it is difficult to obtain satisfactory classification results. Meanwhile, some cancers have been well studied and classified to some subtypes, which are adopt by most researchers. Hence, this priori knowledge is significant for further identifying new meaningful subtypes.

**Results:**

In this paper, we present a combined parallel random forest and autoencoder approach for cancer subtype identification based on high dimensional gene expression data, ForestSubtype. ForestSubtype first adopts the parallel RF and the priori knowledge of cancer subtype to train a module and extract significant candidate features. Second, ForestSubtype uses a random forest as the base module and ten parallel random forests to compute each feature weight and rank them separately. Then, the intersection of the features with the larger weights output by the ten parallel random forests is taken as our subsequent candidate features. Third, ForestSubtype uses an autoencoder to condenses the selected features into a two-dimensional data. Fourth, ForestSubtype utilizes k-means++ to obtain new cancer subtype identification results. In this paper, the breast cancer gene expression data obtained from The Cancer Genome Atlas are used for training and validation, and an independent breast cancer dataset from the Molecular Taxonomy of Breast Cancer International Consortium is used for testing. Additionally, we use two other cancer datasets for validating the generalizability of ForestSubtype. ForestSubtype outperforms the other two methods in terms of the distribution of clusters, internal and external metric results. The open-source code is available at https://github.com/lffyd/ForestSubtype.

**Conclusions:**

Our work shows that the combination of high-dimensional gene expression data and parallel random forests and autoencoder, guided by a priori knowledge, can identify new subtypes more effectively than existing methods of cancer subtype classification.

**Supplementary Information:**

The online version contains supplementary material available at 10.1186/s12859-023-05412-y.

## Introduction

Cancer is a disease closely associated with genetic predisposition, and primarily caused by an imbalance between proliferation and growth-inhibiting apoptosis genes, resulting in abnormal cell proliferation without death [1].

Modern medical research has established that cancer is not a single disease, but rather a collection of hundreds of different diseases. Consequently, cancer can be divided into heterozygous and homozygous cancers. Homozygous cancers can be staged not only according to the stage of cancer development but also according to certain characteristics of the genes in the cancer cells, which allow cancer to be classified into different subtypes [2]. Understanding these cancer subtypes is crucial for developing targeted treatment plans and determining prognosis as cancer subtypes often include valuable information about etiology, cancer biology, and personalized medicine research [3–5]. For one cancer, there maybe have many subtypes, which are significant for treatment. For example, there are currently five traditionally classified subtypes of breast cancer, LumA, LumB, HER2, Basal and Normal, each with different treatment options [6].

Traditional cancer subtype classification may have limitations in implementing precise treatments for patients. Cancers with similar clinical and pathological manifestations may exhibit different behaviors, and identifying targeted and precise treatments based on these different behaviors is the key to treating cancer [6, 7]. To this end, the ability to effectively identify cancer subtypes is crucial for guiding subsequent treatment and improving patient prognosis, making it a meaningful exercise to identify cancer subtypes effectively.

High-dimensional gene expression data can be utilized to analyze changes in gene expression, correlations between genes, and gene activity, among other things. Some cancers have been studied to mark subtype categories, which have been used in many areas of research [8, 9]. Consequently, many cancer subtyping methods use high-dimensional gene expression data to detect cancer subtype.

Currently, various methods for cancer subtype have been presented, which can be categorized into three categories.

1. Methods based on supervised learning. Guo et al. [10] proposed the method BCDForest, which proposes a multi-class granularity scanning method to train the model while finding important features using a new enhancement strategy. Ahmed et al. [11] proposed a cancer subtype classification method using convolutional networks, which mainly uses the ResNext network model and Transformer encoder for feature extraction and classification.
2. Methods based on unsupervised learning. Classification of unlabelled data is more in line with the scope of the clustering problem. Currently, some cancer subtype classification methods use unsupervised learning methods and high-dimensional gene expression data for cancer subtype classification, but the problem is that cancer subtype with no clinical value will be identified when there is no a priori knowledge to guide them. Witten et al. [12] proposed the method SparseK, which uses a lasso penalty to select features and a linear transformation to reduce the dimensionality of the data, and finally SparseK clusters cancer subtype using k-means clustering. Shen et al. proposed the method iCluster [13, 14], which incorporates the associations between different data types and the variance–covariance structure within data types in a single framework, while simultaneously reducing the dimensionality of the datasets. There is matrix inversion involved in this method, so it may have some disadvantages when dealing with high-dimensional data. Monti et al. [15] proposed the method Consensus Clustering, which uses resampling for cancer subtype classification, it provides for a method to represent the consensus across multiple runs of a clustering algorithm and to assess the stability of the discovered clusters. Li et al. [16] select few genes using LASSO and fused three similarity matrices consisting of genes, Iso and miRNA using SKF, and finally clustered the fused similarity matrix with spectral clustering. Nidheesh et al. [17] proposed an improved K-means method, the key idea of which is to select data points that belong to dense regions and are sufficiently separated in the feature space as the initial centroids. In addition, there are methods for joint supervised learning based on prior knowledge guidance. Liu et al. [18] proposed a hybrid depth model, which combines patients’ genetic modality data with image modality data to construct a multimodal fusion framework. Then feature extraction networks are built separately, the outputs of the two feature networks are fused based on the idea of weighted linear aggregation, and finally the fused features are used for prediction. Rather et al. [19] proposed a popular learning based method that uses UMAP and the adaptive noise robust clustering method OTRIMLE to achieve cancer subtype classification.
3. Methods based on a joint supervised learning and priori knowledge. DeepType [20] is the first method to use existing knowledge of cancer subtype to identify new subtype, using known cancer subtype to guide the learning of the model. DeepType also uses deep learning methods for jointly supervised classification, unsupervised clustering and dimensionality reduction.

Although a great deal of research work has been done on the identification of cancer subtype, a number of problems remain in this area:

First, the problem of the “curse of dimensionality” [21], which is characterized by the high dimensionality of gene expression data. Gene expression data typically contain about 20,000 genes, but the number of genes associated with cancer is very small. Therefore, the high-dimensional gene expression data set is sparse, and it is a challenging task to filter out clinically valuable genes to identify subtype. The second feature is the small sample size. Because cancer samples are relatively small, this poses a new challenge to the ability of cancer subtype classification methods to handle small sample datasets.

Second, existing cancer subtype classification methods do not identify new clinically valuable cancer subtype guided by a priori knowledge. Typically, conventional cancer subtype identification methods tend to assign samples to a known subtype or cluster cancer subtype directly without the use of a priori knowledge guidance.

In the face of these two problems, traditional methods of cancer subtype classification tend to select fewer features, and therefore the resulting models are usually prone to bias. How to solve the above problems more effectively will be the focus of this paper.

In this paper, we present a combined parallel random forest and autoencoder approach for cancer subtype identification based on high dimensional gene expression data, called ForestSubtype. Random forest (RF) has advantages in dealing with data sets with small sample sizes and high-dimensional [22, 23]. Moreover, cancer subtypes, which have been known, could be treated as prior knowledge to find features associated with cancer and detect new cancer subtype. For solving the two problem about high dimension and small sample size about gene expression data, ForestSubtype uses a parallel random forest to select significant candidate features based on the priori knowledge of known cancer subtypes. ForestSubtype consider random forest [24] as a base module, and then there are ten random forests executing in parallel, and we call them as a whole as parallel random forest. Note that these ten random forests are independent of each other. Each random forest will get the weight of each feature and rank them. Based on the output of the ten random forests, ForestSubtype gets their intersection of the features with large weight as the candidate features.The parallel random forest can reduce the result randomness compared with a single random forest. And the parallel random forest can obtain the really valuable features, and increase the generalization of the whole model in this paper. After completing the initial dimensionality reduction of the high dimensional gene expression data to select the important gene features, ForestSubtype uses an autoencoder (AE) to further condenses the initial selected gene features into two core features. Finally, k-means++ is used to cluster the cancer samples based on these two core features to identify new cancer subtypes. In this paper, the breast cancer gene expression data obtained from The Cancer Genome Atlas (TCGA) are used for training and validation, and an independent breast cancer dataset from the Molecular Taxonomy of Breast Cancer International Consortium (METABRIC) is used for testing. Additionally, we use two other cancer datasets for validating the generalizability of ForestSubtype. The results show that ForestSubtype outperforms the other two methods in terms of the distribution of clusters, internal and external metric results, and performance comparisons such as validation against independent test sets, achieving more and better results for the identification of new cancer subtypes.

## Methods

**Fig. 1:**
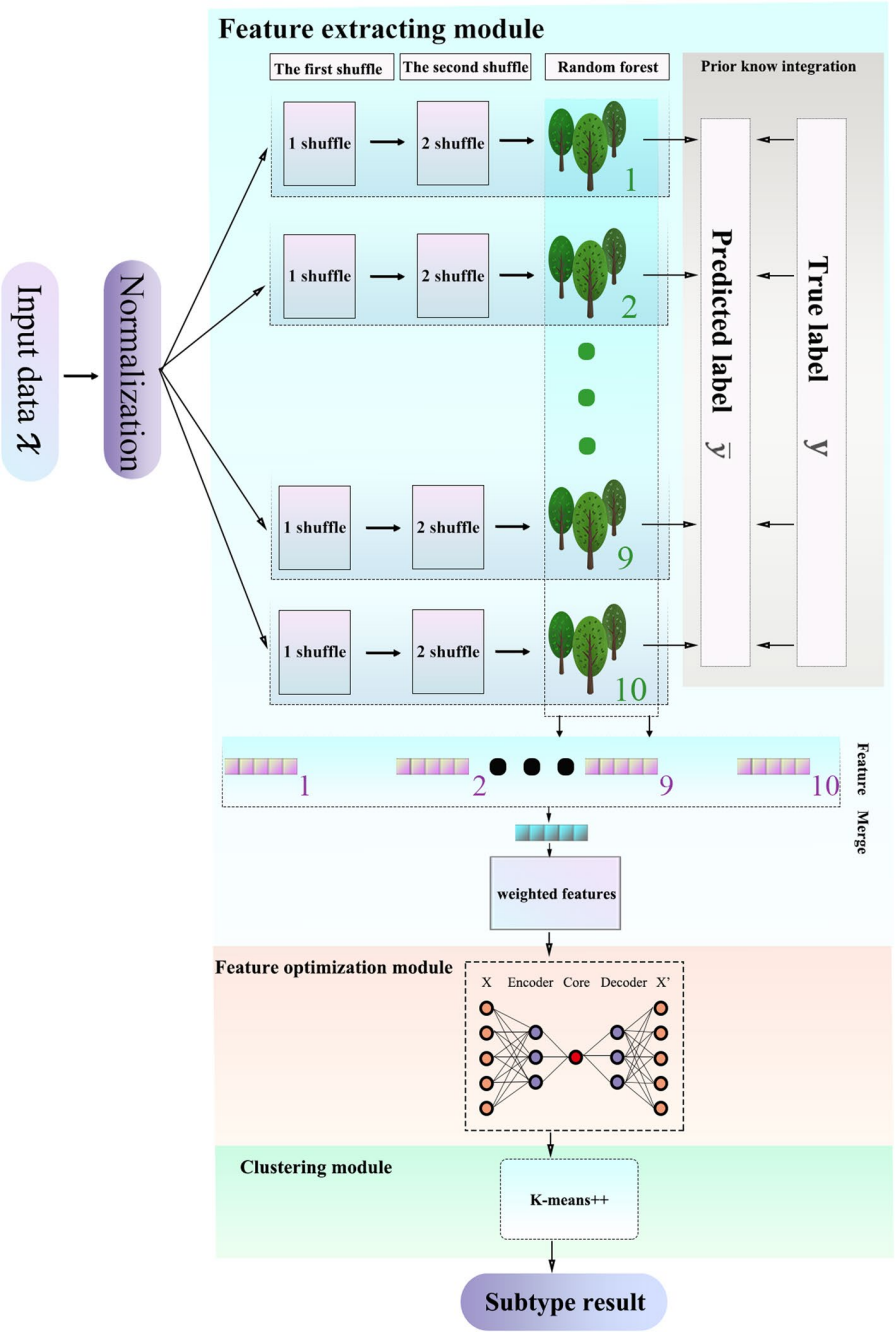
ForestSubtype. It consists of three modules: (i) feature extraction module, (ii) feature optimization module, (iii) clustering module

ForestSubtype adopts high dimensional gene expression data and the prior knowledge about cancer subtype as input. ForestSubtype consists of three modules, which are shown in Fig. 1. (i) Feature extraction module: ForestSubtype first adopts a parallel RF and prior knowledge to train this module and obtain candidate features with high weight. (ii) Feature optimization module: The candidate features output by previous module are further reduced into two core features by an autoencoder module. (iii) Clustering module: ForestSubtype utilizes k-means++ to cluster the final subtypes. We provide a detailed description below.

### Feature extraction module

Let $\mathrm{X}= \left[{x}_{1},{\cdots ,x}_{m}\right]$ be a set of cancer samples, $m$ is the number of the sample, ${x}_{i}$ is the *i*-th sample in $\mathrm{X}$, and Each sample has $C$ genes.$\mathrm{Y}= \left[{y}_{1},\cdots ,{y}_{n}\right]$ is the known subtype as the prior knowledge, $n$ is the number of known prior knowledge subtypes, and ${y}_{j}$ is *j*-th subtype in $\mathrm{Y}$. Let $B$ be a matrix with $m$ rows and $n$ columns. If the *i*-th sample belongs to the *j*-th subtype, ${B}_{ij}$ = 1 else, ${B}_{ij}$ = 0.

ForestSubtype aims to discover new subtypes of cancer and guide the subsequent personalized treatment process. For high-dimensional gene expression data, it is important to select significant features and reduce dimensionality. Furthermore, we should retain the biologically and meaningful features associating with cancer subtypes during the feature extraction step.

Random forest is an ensemble of multiple decision trees, which is an ensemble model. To extract feature results for accuracy, stability, and lower dimensionality, ForestSubtype uses ten random forests for parallel processing. Two kinds of shuffle processing are performed for sample data set, which can guarantee each random forest can output different features. The first shuffle is to disrupt the order of samples, and the second shuffle is a random division of the sample dataset into training set and testing set based on Pareto’s law.

In this module, an RF is a homogeneous integration of all utilized classification and regression tree (CART). In the CART algorithm, its goal is to choose a feature to split samples, which can minimize the cost function (Gini index).

Given a sample set $S$, which is a sampled subset of $\mathrm{X}$, we can calculate the Gini index $Gini\left(S\right)$ of dataset $S$ by Eq. (1).

1 $$Gini(S)=1- \sum_{i= 1}^{n}{({p}_{i})}^{2}$$

where ${p}_{i}$ is the probability that a sample belongs to the *i*-th class, $n$ is the number of the known prior knowledge subtypes.

Suppose sample set $S$ has ${C}^{\mathrm{^{\prime}}}$ features, where ${C}^{\mathrm{^{\prime}}}$ is a subset of feature set $C$. Randomly select a feature $A$ from ${C}^{\mathrm{^{\prime}}}$, then calculate the Gini index of the sample set $S$ under the feature $A$ (see Eq. (2)), and divide the sample set* S* into two parts.

2 $$Gini\left(S,A\right)= \frac{\left|{S}_{1}\right|}{\left|S\right|}Gini\left({S}_{1}\right)+\frac{\left|{S}_{2}\right|}{\left|S\right|}Gini\left({S}_{2}\right)$$

where ${S}_{1}$ is a set which includes the samples whose $A$ is smaller than *a*, where *a* is an value of $A$ in one sample, and ${S}_{2}$ is the remaining samples.

For each feature, this process is performed recursively until a tree is built. The steps of the CART algorithm are as follow:

1. For one sample set, constructing a node including these samples as current node.
2. Calculate the Gini index for each feature and its values of the current node (see Eq. 2).
3. The feature $A$ and its one value *a* with the smallest Gini index is selected as the optimal feature, and the corresponding value *a* is used as the optimal segmentation point.
4. Split the sample set of the current node into two sample subsets. And, the feature $E$ is removed from these two sample subsets. Two new nodes are constructed as left and right nodes of the current node.
5. Then the left and right nodes are processed as current node respectively, and the above steps are repeated.

A random forest randomly collects multiple samples from *X* to form one hundred sample subsets, and uses the above CART algorithm to construct one hundred decision trees. These one hundred decision trees together form a random forest. Based on each decision tree, the random forest uses voting strategy to obtain the final classification results.

Next, we calculate the feature importance of a single random forest using Eqs. (3) and (4). Feature importance refers to the “importance” of each feature. A higher score means that a particular feature will have a greater impact on the model used to predict a sample.

3 $${NI}_{e}= {w}_{e}{Gini(S}_{e})-{w}_{left\left(e\right)}{Gini(S}_{left\left(e\right)})-{w}_{right\left(e\right)}{Gini(S}_{right\left(e\right)})$$

4 $${FI}_{E}= \frac{{\sum }_{e\in R(E)}{NI}_{e}}{{\sum }_{e\in C}{NI}_{e}}$$

where ${NI}_{e}$ is the importance of the node $e$, ${S}_{e}$ is the sample set which includes the samples belonged to $e$. ${w}_{e}$ is the weight of the node $e$, which is the rate of the number of samples in ${S}_{e}$ to the total number of samples. $left\left(e\right)$ is the left child node of $e$, $right\left(e\right)$ is the right child of $e$. ${w}_{left\left(e\right)}$ is the weight of $left\left(e\right)$, ${w}_{right\left(e\right)}$ is the weight of $right\left(e\right)$. This Eq. (3) gives us the importance of $e$. Therefore, we use Eq. (4) to calculate the importance of the feature $E$, where ${FI}_{E}$ is the importance of $E$, $R(E)$ is the node set which include node which is split using the feature *E*.

For each random forest, we select features whose weight is larger *β* (0.001 in default). Then, we can obtain ten feature sets from ten parallel random forests, and next we take the intersection of them (see Eq. 5). Where ${F}_{i}$ represents the features of the *i*-th RF, $F$ is the intersection of the ten feature sets.

5 $$F= \bigcap_{i=1}^{i=10}{F}_{i}$$

The features in *F* is treated as the final features for the following optimization step. We can obtain a new sample set $\overline{X }$ that only contains these 201 features.

## Feature optimization module

Before performing cluster identification, we use an AE [25] module to learn the features in F and condense these features into a two-dimensional data. The AE module contains an input layer, an encoder, a core layer, a decoder, and an output layer. Both the encoder and decoder contain six hidden layers, and the numbers of neurons are symmetric to each other. We use the mean square error function (see Eq. (6)) to measure the error between the original input data x′ and the restored data *f*(*x*′).

6 $$L\left(f\left({\mathrm{x}}{\prime}\right),{\mathrm{x}}{\prime}\right)=\frac{\sum_{i=1}^{Z}{\left(f\left({{\mathrm{x}}{\prime}}_{i}\right)-{{\mathrm{x}}{\prime}}_{i}\right)}^{2}}{Z}$$

where x′ is a sample in $\overline{X }$ and *f*(x′) is the predicted value of x′. $Z$ is the number of the samples in $\overline{X }$. $L$ is the mean square error as loss function. Our optimization goal is to find the lowest loss function value.

Finally, we output the core layer results ${X}_{CL}$ which is a sample set, and each sample in ${X}_{CL}$ is a two-dimension data. We use ${X}_{CL}$ as input for the following classification module.

### Clustering module

ForestSubtype uses the k-means++ [26] (see Eq. (7) and (8)) method to cluster ${X}_{CL}$. This method is based on an improved version of the k-means method, which initializes the centers of clusters away from each other and produces better results than those of random initialization.

7 $$d\left(h,{u}_{i}\right)=\sum_{h\in {cluster}_{i}}{|\left|h-{u}_{i}\right||}_{2}^{2}$$

8 $$D={\sum }_{i=1}^{i=k}d\left(h,{u}_{i}\right),i\in \left(0,\right.\left.k\right]$$

where $h$ is the data belonging to the *i*-th cluster, ${u}_{i}$ is the center of the *i*-th cluster, ${cluster}_{i}$ is the *i*-th cluster, and $d\left(h,{u}_{i}\right)$ is the Euclidean distance between $h$ and ${u}_{i}$. k-means++ randomly selects a data point from each cluster ${cluster}_{i}$ as the new centroid using a weighted probability distribution, where the probability of each point being selected is proportional to its Euclidean distance $d$. The above two steps are repeated until $k$ centers have been selected.

## Results

This section contains six parts: (i) dataset preprocessing; we describe how the input dataset is obtained and processed; (ii) classification method selection; we give a performance comparison between random forest and other classification methods; (iii) experimental parameter settings; we illustrate the parameters in our experiments; (iv) related feature gene results; we discuss the important genes associated with cancer found by ForestSubtype; (v) visualizing subtyping results; we visualize the cancer subtyping results to examine their cluster subtype distributions and the distributions of the prior knowledge labels in the clusters; (vi) performance comparison; first, we validate the training performance between ForestSubtype and the other two competing methods; second, we validate the performance of ForestSubtype and the other competing methods on an independent breast cancer dataset; third, we validate the performance of ForestSubtype on two other cancer datasets.

### Dataset preprocessing

Gene expression data concerning breast cancer are downloaded from the Sangerbox 3.0 platform. The original dataset includes 1211 samples and 56,461 genes. Then, we classify these samples with the genefu package in R. All samples are classified into 5 categories. The genefu package is a PAM50 classification kit. Although this classification process is simple and cannot obtain more sophisticated subtypes, it supplies prior knowledge that can guide the subsequent subtyping step. Note that if one gene is expressed in fewer than 500 samples, it is removed. Finally, we obtain a gene expression dataset containing 1211 samples, each sample includes 23,902 genes, and each sample is labeled. The same steps were followed for the other three datasets, the Molecular Taxonomy of Breast Cancer International Consortium (METABRIC) [27], ACC adrenocortical carcinoma (From TCGA), BLCA uroepithelial carcinoma of the bladder (From TCGA). The information of the four data sets is shown in Table 1.

**Table 1:** Dataset details

Dataset						
*The public breast cancer dataset*						
Label	Basal	Her2	LumA	LumB	Normal	
Num	204	121	198	567	121	
Ratio	17%	10%	16%	47%	10%	
*METABRIC*						
Label	1	2	3	4	5	6
Num	330	239	721	491	202	150
Ratio	15.5%	11.2%	33.8%	23%	9.5%	7%
*BLCA*						
Label	C1	C2	C3	C4	C5	C6
Num	172	90	22	34	91	18
Ratio	40.2%	21.1%	5.2%	8%	21.3%	4.2%
*ACC*						
Label	C1	C2	C3	C4	C5	C6
Num	31	33	5	4	5	1
Ratio	39.2%	42%	6.3%	5%	6.3%	1.2%

### Classification method selection

The feature extraction module is the core of the model. Therefore, we compare the performance of parallel RFs with other five classification methods: k-nearest neighbors (KNN), a support vector machine (SVM), logistic regression, a multilayer perceptron (MLP) and the ensemble method.

For RFs, there are three important parameters: n_estimators, max_depth, max_feature. To avoid costly 3D parameter grid searches, and avoid overfitting, we choose a compromise value of 100 as the actual value of the parameter n_estimators. To balance the decision tree generation time, max_feature is set to be the square root of the total number of features. For max_depth, we perform a grid search between 0 and 20 and evaluate the results with the precision rate metric. For KNN [28], we conduct a grid search between 1 and 20 for the n_neighbors parameter, and the results are evaluated by the precision rate metric. For SVM [29], we perform a grid search between 1 and 10 for the C parameter, and the results are evaluated by the precision rate metric. For logistic regression [30], the saga algorithm is a better choice for a high-dimensional dataset, and its results are evaluated by the precision rate metric. For MLP [31], we use the same parameters as those in DeepType to enable a comparison with this method and then evaluate the results with the precision rate metric. For the combined classifier, we utilize hard voting with two-by-two combinations of the above methods, which are evaluated by a precision rate metric.

**Fig. 2:**
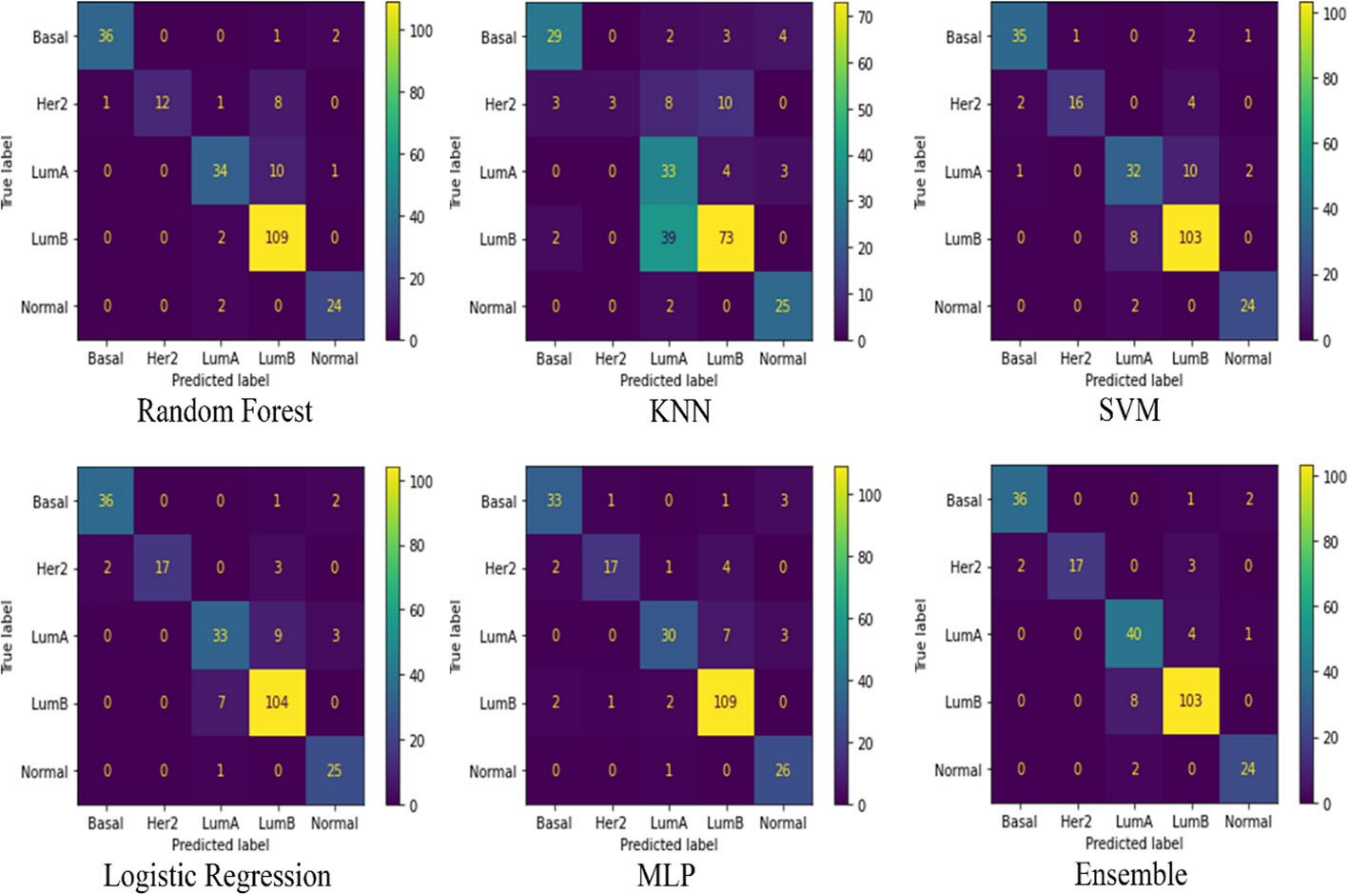
Confusion matrix comparison. It can be observed that RF is higher in classification accuracy compared to the other five methods, and most values are lying on the main diagonal

Table 2 shows the comparison among the precision rates of the six methods (Ensemble, Random Forest, Logistic, MLPClassifier, SVM, K-neighbors), Additional file 1: Table S1 shows the comparison among the F1 and Kappa indices of the six methods, and Fig. 2 shows the confusion matrices of the six methods. After analyzing the precision rates, F1, Kappa, feature extraction effects and confusion matrices, we find that parallel RF has a relatively high precision rate, F1 and Kappa metrics, and the number of values on the main diagonal of the confusion matrix is greater than the number of other regions. Hence, we decide to use the parallel RF in ForestSubtype.

**Table 2:** Comparison of backbone method accuracy rates

	Model	Accuracy
5	Ensemble	0.905350
0	Random Forest	0.884774
3	Logistic	0.884774
4	MLPClassifier	0.872428
2	SVM	0.810700
1	K-neighbors	0.662551

### Experimental parameter settings

The RF is a parallel integrated model with three main parameters, where n_estimators is 100, max_depth is 14 and max_feature is taken as the square root of the original total number of features. For the feature optimization module, we use the AE to condense the features into a two-dimensional feature. The input and output layers contain 201 parameters, the encoder hidden layer contains [1024, 512, 256, 128, 64, 32] parameters, the core layer possesses 2 parameters, the decoder hidden layer includes [32, 64, 128, 256, 512, 1024] parameters, the number of model epochs is 10,000, and the batch size is 128. For the classification module, we use the k-means++ method to find subtypes, with an initialization value of 10 runs, a maximum number of iterations of 300 and a convergence condition of 0.0001.

When using k-means++ to cluster samples, k is an important parameter for clustering result. For obtaining optimal clustering result, we validate different values of k for k-means++. Specifically, we take the value of k in Deeptype as the lower limit and record the silhouette width ${SW}_{Deeptype}$, we then iteratively increase k to achieve the maximum silhouette width and stop when it is greater than ${SW}_{Deeptype}$.

### Related feature gene results

We identified the genes in F that contributed to our identification efforts. From F, we selected 13 representative genes for discussion, and the gene information are obtained from NCBI (National Center for Biotechnology Information) and Google Scholar.

PCAT29: PCAT29 regulates the proliferation, migration and invasion of breast cancer cells and may point to a novel therapeutic target in triple-negative breast cancer [32]. ESR1: This gene encodes a receptor that plays a key role in breast cancer, endometrial cancer and osteoporosis [33]. GATA3: GATA-binding protein 3 (GATA3) have unique clinical implications for breast cancer subtyping and classification [34]. C5AR2: C5AR2 is involved in immune infiltration and malignant characteristics of breast cancer, which may be a prospective biomarker for breast cancer [35]. CCDC170: CCDC170 affects breast cancer apoptosis through IRE1 pathway [36]. FOXC1: a therapeutic biomarker specific for basal-like breast cancer, is not only a potential prognostic candidate but also a potential molecular therapeutic target for this subtype of breast cancer [37, 38]. SLC7A13: The SLC7A family has good diagnostic efficacy in breast cancer [39]. UBE2C: Ubiquitin-binding enzyme E2C (UBE2C) may be oncogenic for the progression of breast cancer genes [40]. SPDEF: SPDEF may play a diversity role in the expression levels, clinicopathologic importance, biological function and prognostic evaluation in BC via bioinformatics and experimental evidence, which mainly depends on different BC subtyping [41]. BIRC5: BIRC5 may be adopted as a promising predictive marker and potential therapeutic target in breast cancer [42]. SPAG5: SPAG5 is a newly amplified gene on Ch17q11.2 in breast cancer and the transcript and protein product of SPAG5 are independent prognostic and predictive biomarkers that may have clinical utility as biomarkers of sensitivity to combination cytotoxic chemotherapy, particularly in estrogen receptor-negative breast cancers [43]. PTTG1: PTTG1 may increase breast cancer (BC) cell growth through nuclear exclusion of p27, highlighting a novel molecular regulatory mechanism in breast cancer (BC) tumorigenesis [44].

### Visualizing subtyping results

**Fig. 3:**
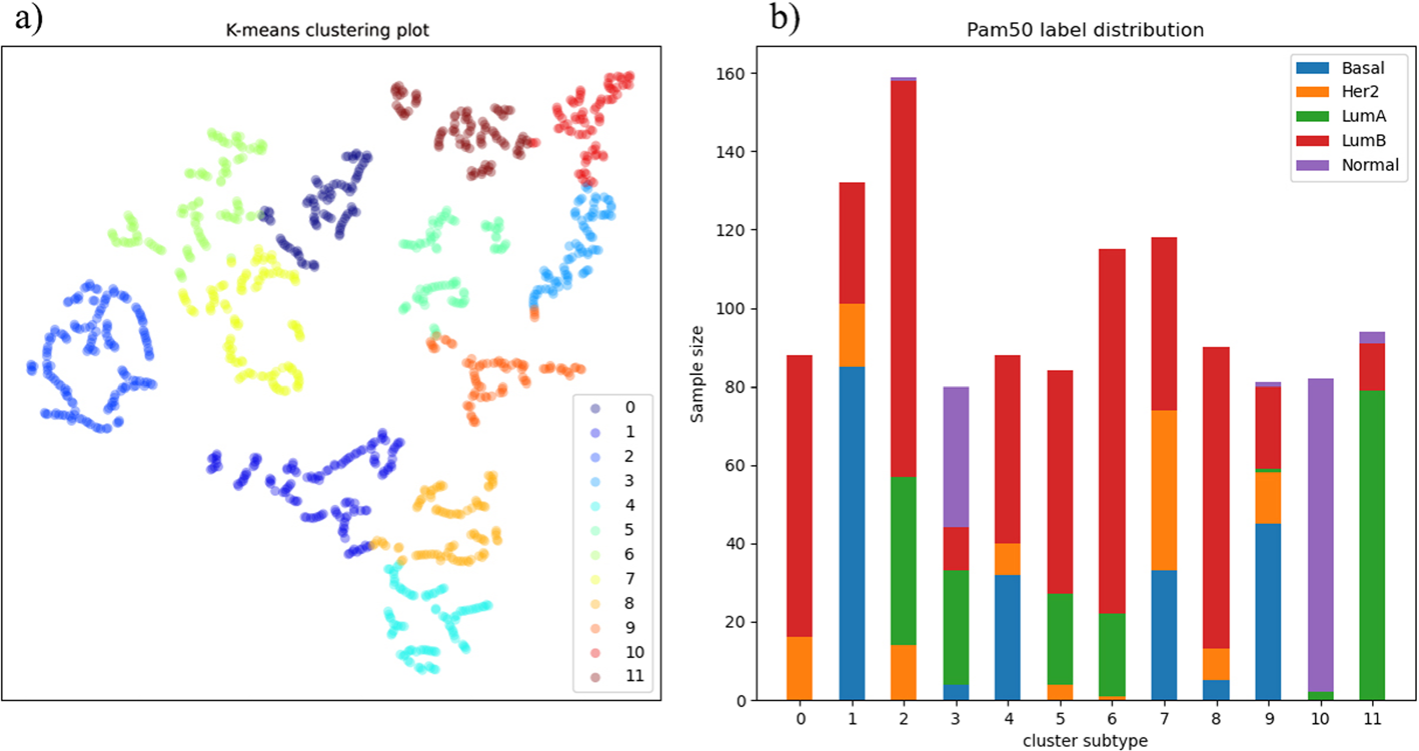
Visualizing Subtyping results. **a**, **b** represent the distributions of the identified clusters; we can see from (**a**) that the samples are divided into 12 clusters with labels 0–11. As shown in (**b**), we can see that the label 10 is almost normal; LumB is the majority in labels 0, 2, 5, 6 and 8; LumA is the majority in label 11; Her2 is mostly distributed in label 7; and Basal is mostly distributed in label 1

We apply ForestSubtype to the public breast cancer dataset and detect 12 subtypes. Visualizing the high-dimensional clustering subtype results allows us to intuitively feel the effect of the model and the distribution of data for each cluster. To visualize the high-dimensional cluster subtype results, we use the t-SNE method [45] to visualize the high-dimensional manifold data in a low-dimensional space. Figure 3a, b represent the distributions of the identified clusters; we can see from Fig. 3a that the samples is divided into 12 clusters with labels 0–11. As shown in Fig. 3b, we can see that label 10 is almost normal; LumB is the majority in labels 0, 2, 5, 6 and 8; LumA is the majority in label 11; Her2 is mostly distributed in label 7; and Basal is mostly distributed in label 1. We also note that in Fig. 3b, there is a certain amount of inconsistency between the cancer subtype labels and the prior known labels. This phenomenon is normal, as the method in this paper is developed based on prior knowledge (known subtypes) to obtain new subtypes.

### Performance comparison

For validating the performance of ForestSubtype, we compared it with other two methods DeepType [20] and SparseK [12].

Firstly, we train and test the three methods on the public breast cancer dataset to compare their performance.

**Fig. 4:**
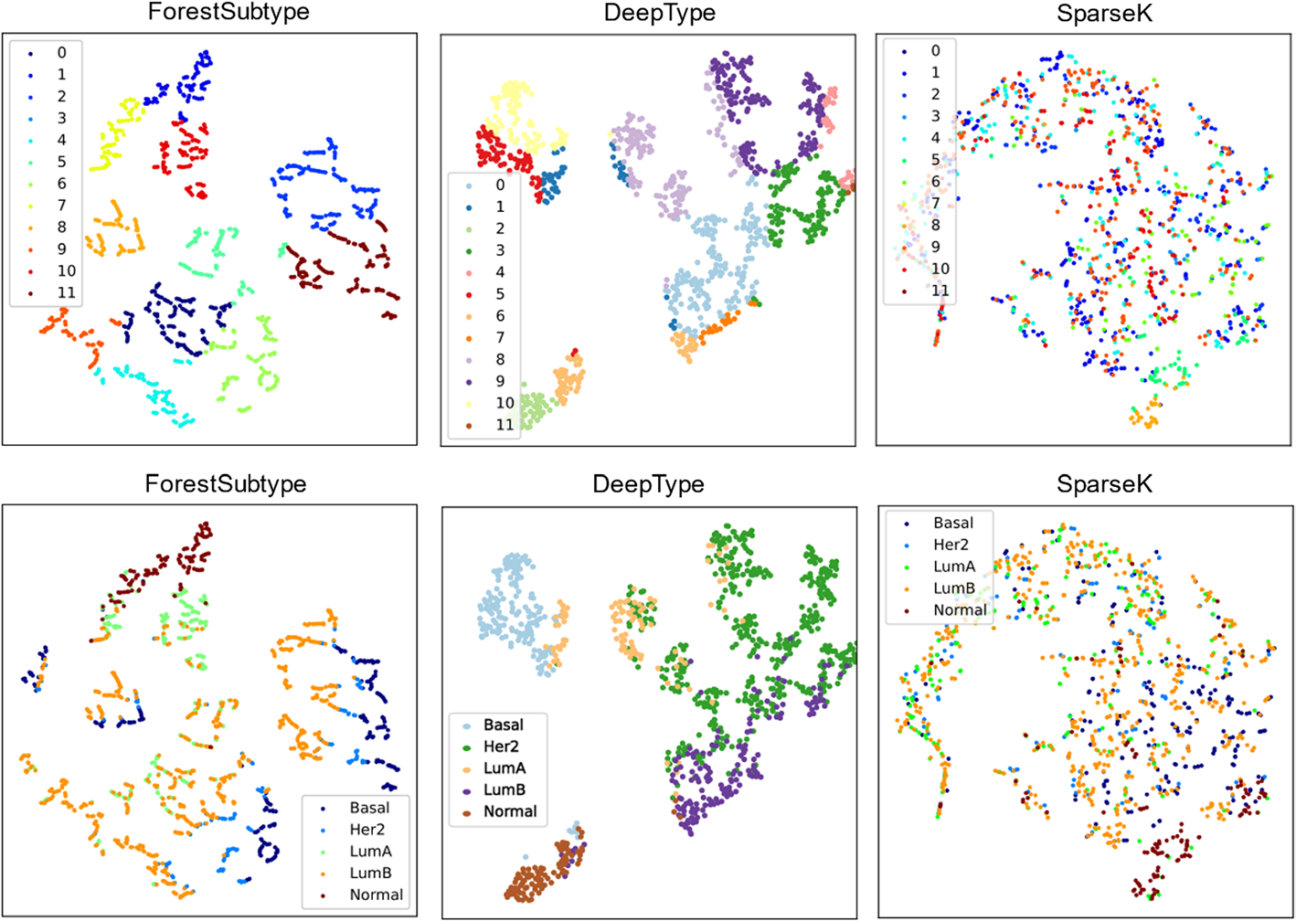
Cluster distribution. The three top figures are distributions of clusters detected by three methods respectively. The three below figures are distribution of prior known subtypes

We divided the public breast cancer dataset into two subsets of 80% and 20% according to Pareto’s law. We used the former for training ForestSubtype and the other two competing methods Deeptype and SparesK, and used the latter for testing these three methods. To compare the advantages and disadvantages of the three models, we first visualize the subtype results of the three methods using the t-SNE method, as shown in Fig. 4. We can see that ForestSubtype identifies 12 subtypes with clear boundaries. We also find that the normal samples are almost distributed together, which is consistent with the a priori knowledge distribution. Looking at the cluster distribution of the other two methods, the boundary of clusters detected by DeepType is not very clear. The clusters detected by SparseK is chaotic and the clustering structure is not identifiable. By analysis of the results, ForestSubtype has a clear clustering structure compared to the other two methods, but the samples in one cluster are less tightly packed. We then test the three methods using internal and external evaluation metrics. For the external metric PAM50, we measure the results using the mean purity [46] and normalized mutual information (NMI) [47], both of which assess the similarity of the clustering results to the true state of the dataset. Both the mean purity and NMI takes a value between 0 and 1, with higher values indicating a higher degree of similarity between the clustering results and the true state of the dataset. The specific implementation of the average purity is shown in (Eq. 9).

9 $$Purity(\Gamma ,\Delta )= \frac{1}{N}\sum_{i}\underset{j}{\mathrm{max}} \left|{\gamma }_{i}\cap {\delta }_{j}\right|$$

where $\Gamma$ is the set of clustering results, $\Delta$ is the true state of the dataset, ${\gamma }_{i}$ is all samples in the *i*-th cluster, ${\delta }_{j}$ is the true sample in the *j*-th category, $N$ is the total sample size. The results of the external metrics are shown in Table 3, and through the results, we find that ForestSubtype outperforms the other two methods. We next measure the three methods using two internal evaluation metrics, the silhouette widths [48] and Davies-Bouldin index (DBI) [49], both of which assess the cluster quality of the methods in terms of compactness and separability (compactness represents the compactness within the same cluster, while separability means the separability between different cluster). The silhouette widths takes a value between -1 and 1, with higher values indicating better compactness within the same cluster and better separability between different clusters. The specific implementation of the silhouette widths is shown in (Eq. 10).

10 $$SW= \frac{1}{N} \sum_{i=1}^{N}\frac{{\eta }_{\mathrm{i}}- {\zeta }_{i}}{max({\zeta }_{i},{\eta }_{i})}.$$

where ${\zeta }_{i}$ is the average distance between the *i*-th sample and the other samples in its same cluster, ${\eta }_{\mathrm{i}}$ is the average distance between the *i*-th sample and the nearest sample in the different clusters, $N$ is the total sample size. DBI takes a value between 0 and 1, with lower values indicating better compactness within the same cluster and better separability between different clusters. The specific implementation of DBI is shown in (Eq. 11).

11 $$DBI= \frac{1}{k} \sum_{i=1}^{k}\underset{j\ne i}{\mathrm{max}}\frac{\overline{{\Omega }_{i}}+ \overline{{\Omega }_{j}} }{{{||\Psi }_{i} - {\Psi }_{j}||}_{2}}.$$

where $\overline{{\Omega }_{i}}$ is the average Euclidean distance from the sample of the *i*-th cluster to its cluster center, $\overline{{\Omega }_{j}}$ is the average Euclidean distance from the sample of the *j*-th cluster to its cluster center, ${{||\Psi }_{i} - {\Psi }_{j}||}_{2}$ is the Euclidean distance between the cluster centers of the *i*-th and *j*-th clusters, $k$ is the number of clusters. The results of the internal evaluation metrics are shown in Table 4, and through the results, we find that ForestSubtype is optimal among the three methods.

**Table 3:** Average purity and NMI comparison

	Average purity	NMI
ForestSutype	0.82	0.60
DeepType	0.72	0.51
SparseK	0.63	0.38

**Table 4:** Silhouette width and DBI

	Silhouette width	DBI
ForestSutype	0.470	0.721
DeepType	0.311	0.913
SparseK	0.214	1.786

In summary, the proposed method has cancer subtyping results with higher quality than those of the other two methods.

Next, we further verify the performance of the three methods on an independent breast cancer dataset.

**Fig. 5:**
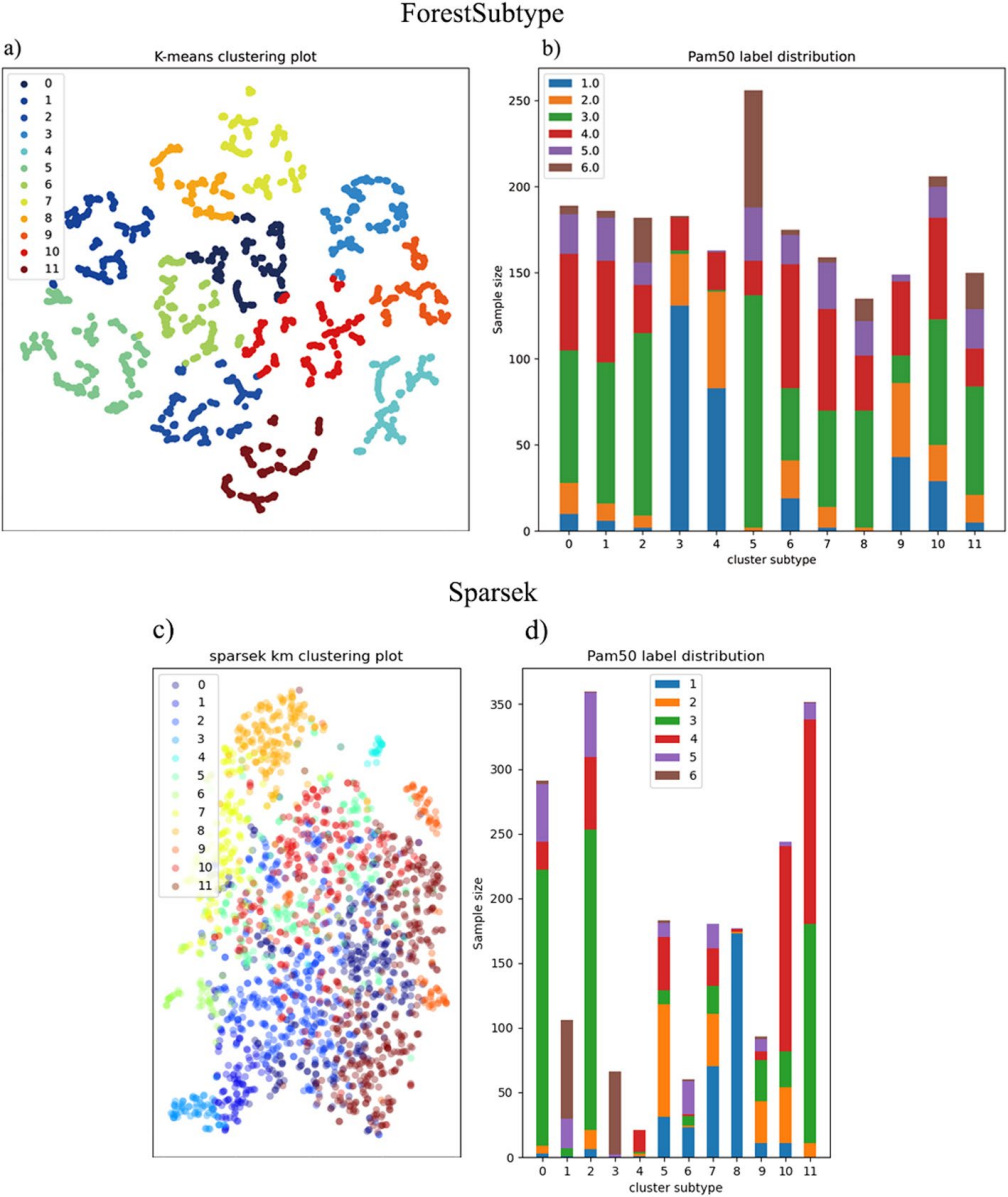
Subtyping result on METABRIC dataset. From **a**, **b**, it can be seen that ForestSubtype can also be divided into 12 clusters on the test set. From **c**, **d** it can be seen that SparseK cannot be divided into clear clustering structures

We use the public breast cancer dataset preprocessed in “Dataset preprocessing” section for training and the Molecular Taxonomy of Breast Cancer International Consortium (METABRIC) dataset for testing [27]. The METABRIC dataset contains 2133 samples and 20,000 gene features. Due to the different gene feature dimensions in the two datasets, situations may occur in which the selected feature genes are not found in the test set. Therefore, we take the intersection of the features in the two sets, and obtain a training set with 12,855 feature genes and a test set with 12,855 feature genes, where the former (the training set) is a subset of the dataset introduced in “Dataset preprocessing” section and the latter (the test set) is a subset of the METABRIC dataset. We first train the model on the training set and then conduct a clustering on the test set to determine the 12 clusters, and the results are shown in Fig. 5a, b. From the results, we can observe that the distribution of clusters are clear and easily identifiable boundaries. In addition, we calculate a p value [50] for the results, and it shows that the identified clusters are significant. The results for SparseK are shown in Fig. 5c, d, where we can see that the method does not have a clear clustering structure.

In summary, the proposed method generalizes well to the test set.

Finally, we validate the performance of the proposed method on other types of cancer datasets.

**Fig. 6:**
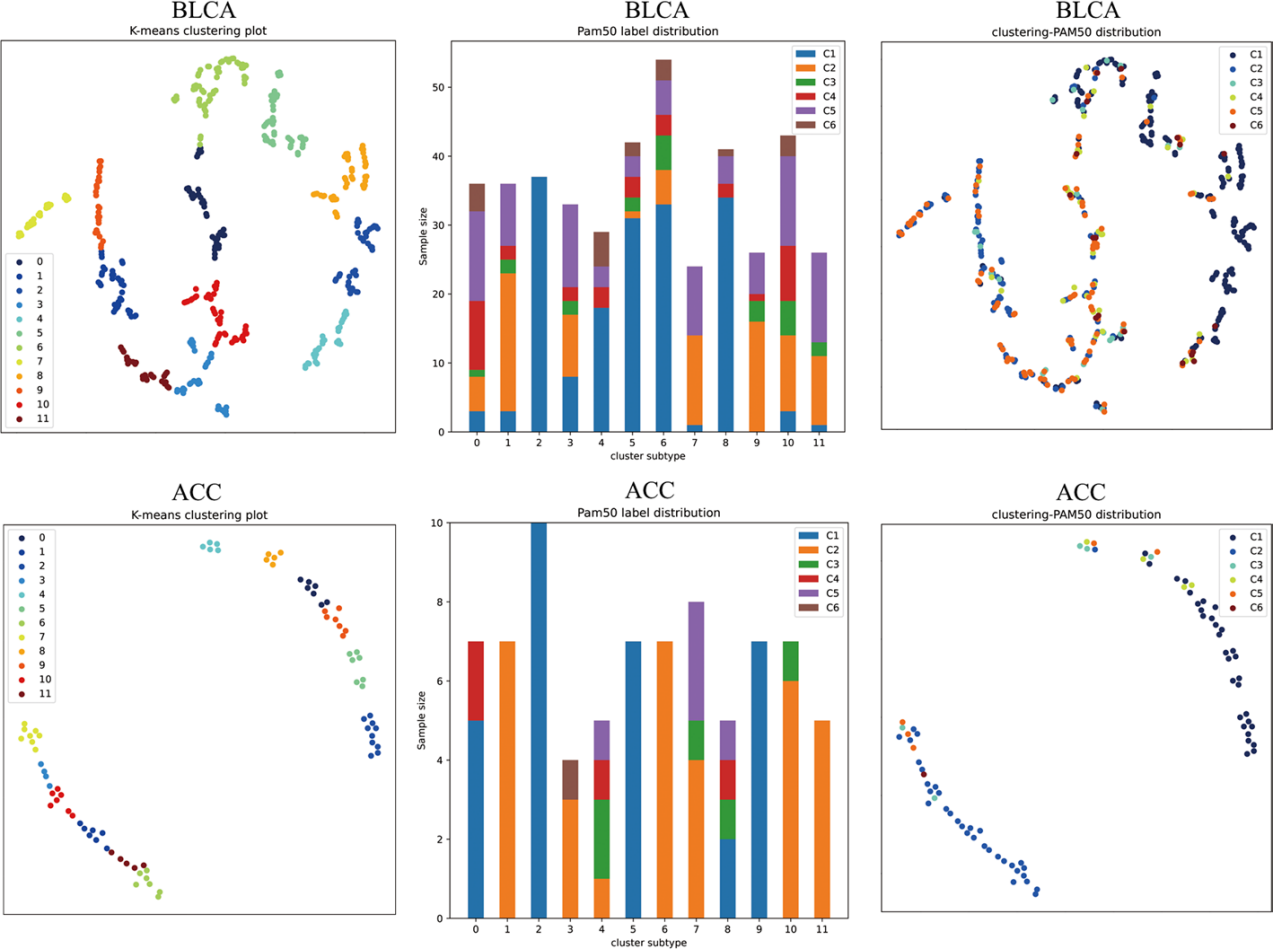
Comparison of subtypes of heterogeneous cancers. The top shows the distribution of BLCA clusters and a priori space labels, while the bottom shows the distribution of ACC clusters and a priori space labels

We select ACC adrenocortical carcinoma with a small sample size and BLCA uroepithelial carcinoma of the bladder with a large sample size from TCGA database to test the ability of ForestSubtype. After performing the steps described in “Dataset preprocessing” section, the gene expression data of both cases are obtained (where the ACC dataset has 79 samples and 257,769 gene features, and the BLCA dataset has 427 samples and 28,290 features). In addition, we form the corresponding prior knowledge subtypes on the ACC and BLCA datasets by the method described in the PAM50 paper [51]. Two other types of cancer datasets are constructed. Then, the two cancer datasets are trained and tested by the method proposed in this paper, and the test results are shown in Fig. 6. We find that the samples in the BLCA dataset are divided into 12 cancer subtypes with clear and easily identifiable cluster boundaries. In contrast, the results obtained on the ACC dataset with a relatively small sample size are not satisfactory, because the small sample size of the ACC dataset causes feature overlearning, resulting in poor model performance. The small sample size means that there are not enough samples in the training set to cover the entire data space, and the high dimensionality means that each sample has many features, making the sample space more sparse, which will increase the fitting error of the model on the training set. Overfitting, on the other hand, results in reduced performance of the model on the test set, as the model has overfitted to the noise or randomness in the training set and is unable to generalize to new data sets. For this problem of the ACC dataset, we first performed the data augmentation process on the ACC dataset and then repeated the previously described steps on the augmented dataset. Two methods were used in the data augmentation part for the comparison study, they are SMOTE [52] and Borderline SMOTE [53], the details are shown in Additional file 1: Figs. S1 and S2.

## Discussion

This paper proposes a cancer subtyping method, named ForestSubtype, based on a parallel RF and autoencoder, which uses the prior knowledge and high-dimensional gene expression data to obtain new subtypes. First, some significant candidate features are extracted by ForestSubtype based on the priori knowledge of cancer subtype. Second, the features with large weights are selected. Third, ForestSubtype uses an autoencoder (AE) to condenses the selected features into a two-dimensional data. Fourth, ForestSubtype utilizes k-means++ to obtain the final clustering results. Our experiments demonstrate that ForestSubtype have a better performance than other two methods. In this paper, we only focus on the gene expression data, but some other types of data (DNA methylation dataset) may play a important role in the mechanism of cancer subtyping [54]. In the future, we will combine the gene expression and DNA methylation data to study cancer subtype.

## Conclusions

In this paper, we propose a parallel RF and autoencoder based cancer subtype identification method, named ForestSubtype, which uses prior knowledge and high-dimensional gene expression data to obtain new subtypes. Our work shows that the combination of high-dimensional gene expression data and parallel random forests and autoencoder, guided by a priori knowledge, can identify new subtypes more effectively than existing methods of cancer subtype classification. This paper focuses on only one dimension, gene expression. In the future, we will combine the gene expression and DNA methylation data to study cancer subtype.

## Supplementary Information

**Additional file 1**. Supplementary materials of ForestSubtype.

## Data Availability

The original data for the METABRIC dataset was obtained from http://www.acsu.buffalo.edu/~yijunsun/lab/DeepType.html. The raw data for ACC, BLCA and the breast cancer training set used in this paper were obtained from https://www.aliyundrive.com/s/9kzrVjtKLFU. Project name: ForestSubtype. Project home page: https://github.com/lffyd/ForestSubtype. Operating system(s): Linux or other unix-like systems. Programming language: python 3.x. Other requirements: R. License: GNU GPL v3. Any restrictions to use by non-academics: license needed.

## Abbreviations

RF: Random forest
TCGA: The Cancer Genome Atlas
METABRIC: Molecular Taxonomy of Breast Cancer International Consortium
AE: Auto Encoder
CART: Classification and regression tree
KNN: K-nearest neighbors
SVM: Support vector machine
MLP: Multilayer perceptron
NCBI: National Center for Biotechnology Information
BC: Breast cancer
NMI: Normalized mutual information
DBI: Davies-Bouldin index
ACC: Adrenocortical carcinoma
BLCA: Bladder urothelial carcinoma
