# Supplementary material for: ForestSubtype: a cancer subtype identifying approach based on high-dimensional genomic data and a parallel random forest
Source: BMC Bioinformatics. 2023 Jul 19;24:289. doi: 10.1186/s12859-023-05412-y (PMC10354904; doi:10.1186/s12859-023-05412-y)
Supplement: Supplementary file 1 — Additional file 1. Supplementary materials of ForestSubtype. [file 12859_2023_5412_MOESM1_ESM.docx]

**Supplementary Materials**

ForestSubtype: A cancer subtype identifying approach based on high-dimensional genomic data and a parallel random forest

**1 Evaluation Experiment on Classification Methods**

In Section 3.2 of the main text, all methods are evaluated by F1 and Kapa. The results of these two metrics are shown in Table S1.

**Table S1.** Comparison of classification methods

|  | Model | F1 | Kappa |
| --- | --- | --- | --- |
| 5 | Ensemble | 0.865562 | 0.866675 |
| 0 | Random Forest | 0.856114 | 0.832731 |
| 3 | Logistic | 0.854210 | 0.836368 |
| 4 | MLPClassifier | 0.842105 | 0.819500 |
| 2 | SVM | 0.801453 | 0.786451 |
| 1 | K Neighbors | 0.566826 | 0.537637 |

**2 Supplemental Experiments on Sample Size Statistics before and after ACC Dataset Augmentation**

In Section 3.6 of the main text, the ACC dataset contained 79 samples and 257,769 gene features prior to data augmentation. The statistical results before augmentation are presented in Fig. S1 a). Label C6, which had a sample size of 1, was excluded prior to augmentation. Two data augmentation methods, namely SMOTE and Borderline SMOTE, were employed in this experiment, and the statistics after augmentation are shown in S1 b) and S1 c), respectively. The ACC dataset augmented by both methods was then fed into the model proposed in this paper, and the experimental results are shown in Figure S2. The top part of Figure S2 presents a map of the cancer subtype classification after augmentation with SMOTE, while the bottom part presents a map of the cancer subtype classification after augmentation with Borderline SMOTE. As depicted in Figure S2, the data-augmented ACC classification results are somewhat better than the data unaugmented ACC classification results presented in Figure 6. Moreover, when comparing the results of SMOTE and Borderline SMOTE, SMOTE produced somewhat better results.


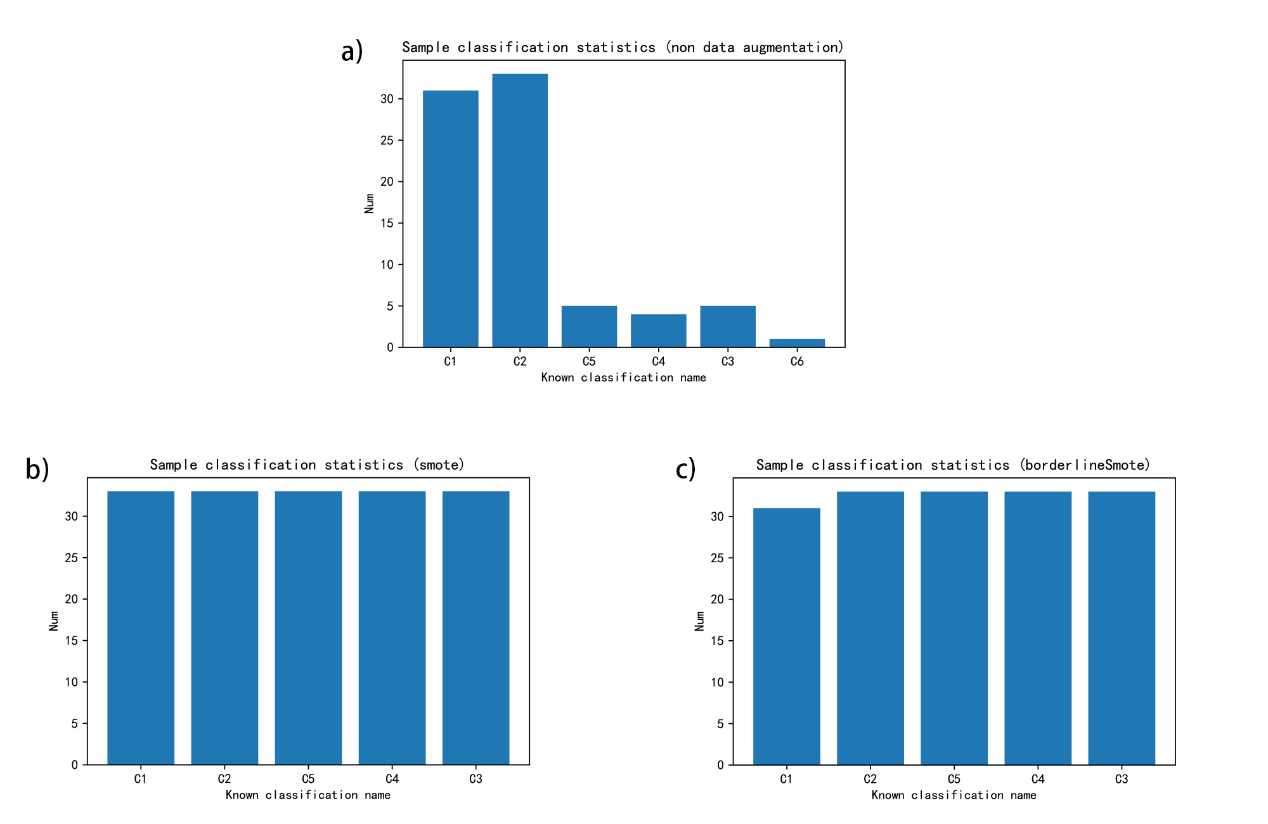


**Fig. S1 Statistical comparison before and after data augmentation.** This graph shows the sample size statistics before and after data augmentation. Where a) is the sample size statistics before data augmentation yet, b) is data augmentation using SMOTE method, and c) is data augmentation using Borderline SMOTE.


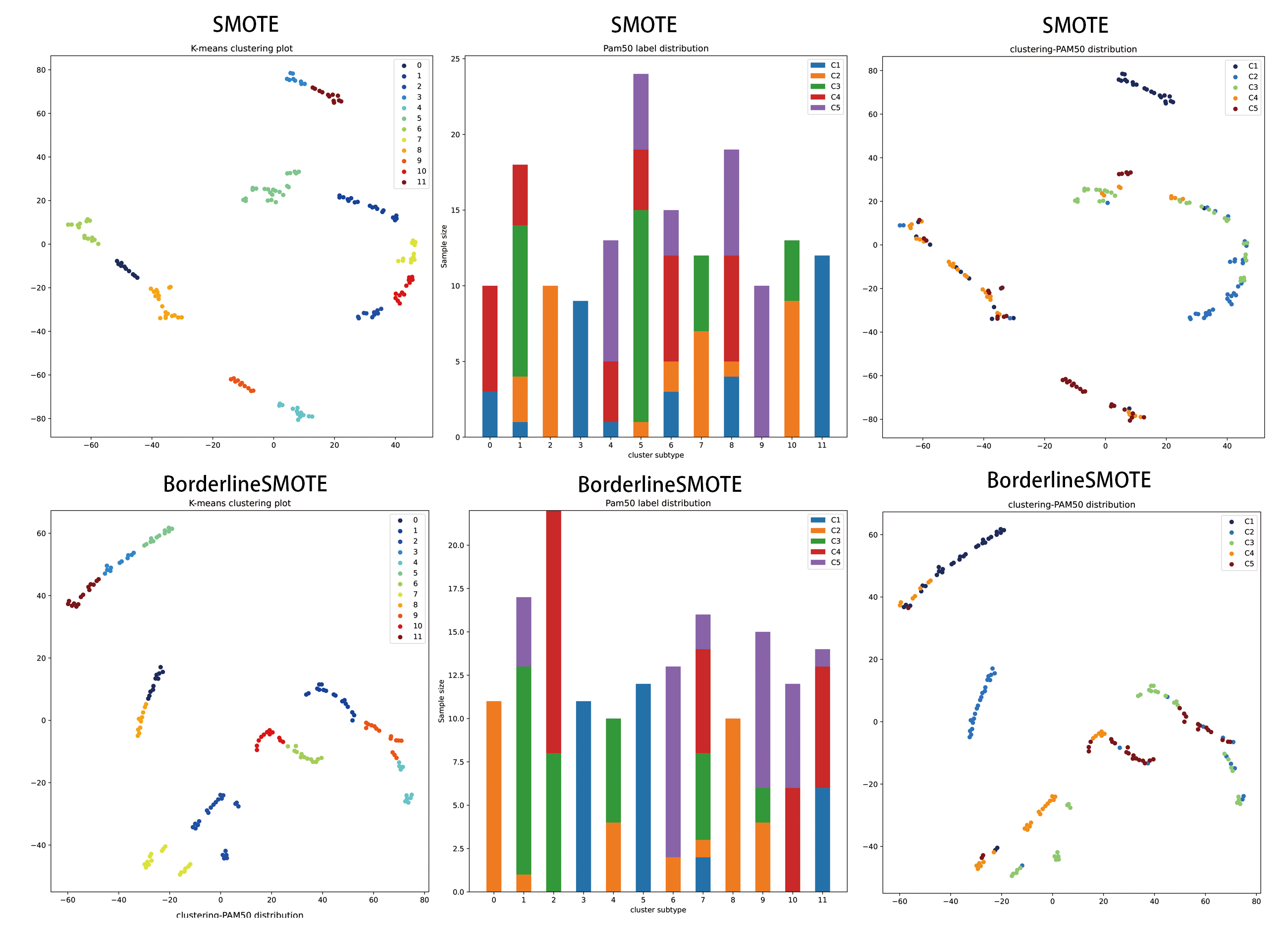


**Fig. S2 Comparison of ACC subtypes for different data augmentation methods.** The distribution of ACC clusters and a priori spatial labels after augmentation with SMOTE is shown above, and the distribution of ACC clusters and a priori spatial labels after augmentation with Borderline SMOTE is shown below.

**3 Comparison experiment with and without autoencoder**

To assess the utility of the autoencoder, we created two versions of ForestSubtype: one with the autoencoder and one without. The former is referred to as ForestSubtype (AE), while the latter is referred to as ForestSubtype (Without AE). The left half of Figure S3 displays the cancer subtype visualization results of ForestSubtype (AE) along with its prior knowledge distribution results, while the right half displays the cancer subtype visualization results of ForestSubtype (Without AE) along with its prior knowledge distribution results. We visualized the cancer subtype results identified by both models, which are presented in Figure S3. We evaluated the clustering performance of both models using both internal evaluation metrics (Silhouette width, DBI) and external evaluation metrics (Average purity, NMI). The external evaluation metrics results are presented in Table S2, and the internal evaluation metrics results are presented in Table S3. As evident from Figure S3 and Tables S2-S3, the evaluation results of ForestSubtype (AE) are superior to those of ForestSubtype (Without AE). Therefore, the autoencoder enhances the model's ability to identify cancer subtypes.


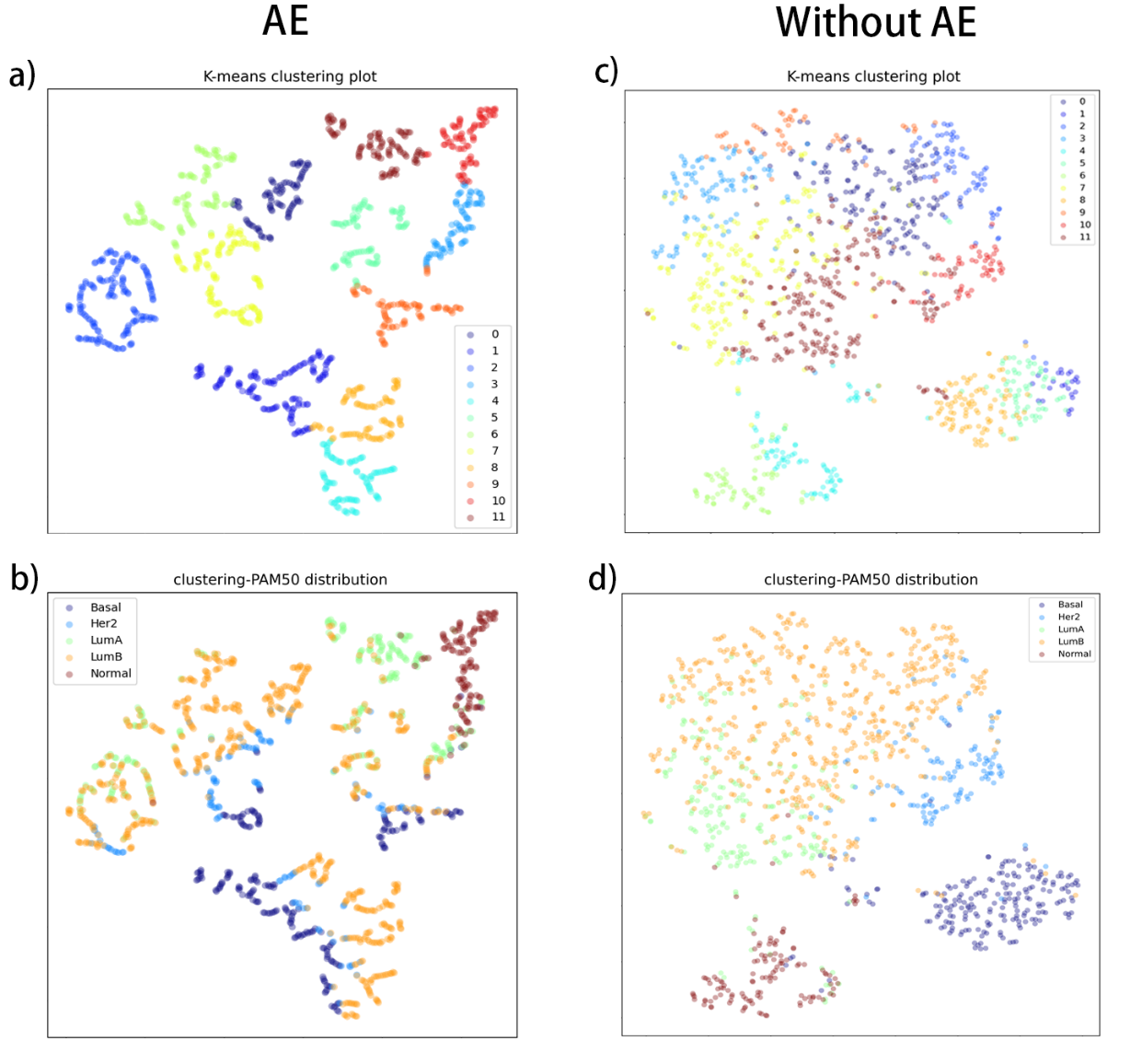


**Fig. S3 Comparison visualization with and without auto encoder.** Figure S3(a-b) shows the visualization results of the model including the autoencoder, a) the results of the identified cancer subtypes, and b) the corresponding prior knowledge distribution. Figure S3(c-d) shows the visualization results of the model without the autoencoder, c) is the result of the identified cancer subtypes, and d) corresponds to the a priori knowledge distribution.

**Table S2.** Average purity and NMI comparison

|  | Average purity | NMI |
| --- | --- | --- |
| ForestSutype (AE) | 0.82 | 0.60 |
| ForestSutype (Without AE) | 0.65 | 0.42 |

**Table S3.** Silhouette width and DBI

|  | Silhouette width | DBI |
| --- | --- | --- |
| ForestSutype (AE) | 0.470 | 0.721 |
| ForestSutype (Without AE) | 0.044 | 2.446 |
